# The Possibility of Open-Label Use of Placebo in Healthcare Practice: A Systematic Review of Recent Clinical Trials

**DOI:** 10.7759/cureus.95655

**Published:** 2025-10-29

**Authors:** Frederick, Chin Wang Tao, Faisal Ghafoor, Noel Yang

**Affiliations:** 1 Sixth Form, Brighton College, Brighton and Hove, GBR; 2 Pain Management, The Superior University, Lahore, PAK

**Keywords:** a systematic review, clinical trials, healthcare practice, open-label placebo, open-label placebos (olps)

## Abstract

The use of placebos in primary treatment causes ethical concerns, as some may argue that the practice of prescribing placebos is deceptive. As a result, patients must be well-informed about their treatment plan and medications for physicians to maintain a positive patient-care relationship. This literature review evaluates the data from clinical open-label placebos (OLPs) and proposes preliminary suggestions regarding why the practice of prescribing placebos without deception may be effective. This systematic review aimed to address the research question: With patients receiving healthcare, if introduced, do OLPs improve patient-related or objective functional outcomes compared with the methods of usual care (UC) or treatment as usual (TAU)?

A systematic review was conducted following Preferred Reporting Items for Systematic Review and Meta-Analysis (PRISMA) 2020 guidelines. The keywords “open-label placebos (OLPs), patient-related outcomes, functional outcomes, UC, TAU, and clinical trials” were used to search for studies from PubMed, Cochrane Library, and Scopus. The search was limited to articles written in English and published between the 1st of January 2020, and before the 31st of August 2025. The risk of bias (RoB) was assessed using Cochrane RoB 2.0. A narrative approach was used to synthesize evidence due to the heterogeneity of the studies.

The review retrieved 120 articles initially. As not all studies are relevant, only 15 randomized controlled trials (RCTs) (nine of them with some concerns and six with low RoB) were included to synthesize evidence.

OLPs are a potentially ethically viable intervention, as they effectively impact subjective symptom perception without changing the pathological discourse of the underlying disease. It works effectively, especially when it comes to symptom management of chronic conditions with a dominating subjective factor (e.g., patient- and psychosomatic-related symptoms or complications). OLPs do not meaningfully change the pathological course of the disease. Its effectiveness is seemingly due to a complex interaction between clear communication with patients and the ability for patients to regulate the perception of the symptoms. Therefore, OLPs should not be used as a standalone treatment to replace evidence-based primary treatment of clinical, chronic conditions, but OLPs can very possibly be considered as an add-on treatment if current primary treatments have failed, were ineffective, or were limited or have side effects that are intolerable. OLPs can be prescribed to patients who do not wish to proceed with primary treatment. The methodological challenges need to be addressed by future researchers.

## Introduction and background

Over the past two decades, the practice of using placebos as a method to reduce bias has become more standardized in legislation and various medical standards. As a result, placebos have been studied extensively, with an increasing number of clinical trials using placebos as an intervention. The included literature shows that inert medications significantly lessen symptoms [1]. Outside of clinical studies, placebos are already widely employed in medical practice [2]. In the United Kingdom (UK), 77% of general physicians (GPs) use open-label placebo (OLP) due to their low cost and lack of pharmacological adverse effects [3].

The use of traditional placebos in primary treatment creates ethical concerns, as some might hold the view that doctors are prescribing deceptive drugs. Therefore, patients must be well informed about their treatment plan and medications for physicians to maintain a positive patient-care relationship [4]. Despite ethical concerns, some researchers believe deceptive placebos are permissible in some cases, as the therapeutic interaction can still benefit the patient, adhering to the pillar of beneficence. Some claim that doctors use deception in order to bring about positive outcomes to get better treatment outcomes, which could impair the doctor-patient relationship [5]. This question concerns whether placebo effectiveness will be impaired if patients are aware that the medication they are given is a placebo instead of an active drug. To find out, this paper is focused on how OLP will impact patients. OLP is defined as any type of placebo intervention in which patients are clearly told that they are receiving a placebo, and the placebo is given in any form (oral capsule, solution, spray, injection, sham device, or mouthwash). The main objective of OLP is to make use of the placebo effect without deceiving their patient. OLP may incorporate conditioned open-label placebos (C-OLP), which denote the combination of a placebo with psychological conditioning (e.g., behaviors such as expectation or prior associations to enhance the placebo effect). The most important criterion is that the patient knows that he or she is receiving a placebo even when psychological conditioning of additional types or different kinds of delivery are involved. 

Recent reviews evaluated clinical OLP data and proposed preliminary suggestions for why OLPs prescribed without deception may be effective [6,7]. The main challenge in placebo treatment studies is distinguishing between a placebo effect and a placebo response. Alongside the placebo effect itself, several factors can influence patient outcomes, which can include the natural tendency of some conditions to improve over time and the Hawthorne effect, where the patients are getting extra attention during the study and perform better. Blease et al. identified three key methodological challenges of OLP research: control group selection, lack of blinding, and teaching/explaining of OLP information for patients. Most OLP studies involve comparisons of the intervention and treatment as usual (TAU) or no treatment (NT) controls, such as a waitlist. However, critics believe that patients in TAU or NT groups may not always receive as much engagement or follow-up as patients in the OLP group, so it is difficult to be sure that both groups receive the same treatment [6]. This difference in attention can create bias; the OLP group may improve due to increased attention (Hawthorne effect) [7], while the control patients may feel they have been neglected or disappointed [8], and this may worsen their outcome (nocebo effect) [9]. Another issue is blinding. As OLP participants are told they are getting a placebo, neither the patient nor the clinician can be blinded to the intervention. There is a chance of bias. If outcome assessors are blinded and OLPs are embedded in well-controlled pharmacological trials using standard procedures of informed consent, it can help to reduce bias [10].

OLP's administration instructions and narratives to patients matter. In most OLP trials, the administrator explained the pill's inactive, inert nature and explained the advantages of taking the placebo and encouraged patients by saying many have benefited from a placebo [11-13]. One study has shown that placebos without deception can have beneficial effects [14]. Experimentally, some non-clinical research explored the OLP effect and its dependence on different instructions. These studies imply that a story that raises positive expectancies in participants, even when the inert nature of the medicine is explained, is critical to generating positive OLP effects.

However, this field is witnessing growth in research trials. Strangely, the current state of OLP research reports limited articles, which emphasize conducting more systematic reviews, including recent trials from the last five years, to incorporate updated knowledge and observe the impact of OLP with or without informed prescription. This systematic review aimed to address the research question: In patients receiving healthcare, if introduced, do OLPs improve patient-related or objective functional outcomes compared with usual care (UC) or TAU? The evidence provides a comprehensive review of efficacy, outcomes comparison (patient-related versus objective outcomes), methodological limitations, and ethical implications of OLP.

## Review

Methodology

Study Design

A systematic review was conducted following the Preferred Reporting Items for Systematic Review and Meta-Analysis (PRISMA) 2020 guidelines [15].

Population, Intervention, Comparison, Outcome, Study, Time (PICOST) Framework

The following text words and keywords: “Open-label placebo,” “Patients at clinical healthcare settings,” “Usual care (UC),” “Treatment-as-usual (TAU),” “Standard medical management,” “Symptom relief,” and “Safety outcomes” were used to construct the research question using the PICOST framework (Table 1).

**Table 1:** PICOST framework OLP: open-label placebo; RCTs: randomized controlled trials

PICOST Framework	Text Words (Domains)
P (Population):	Patients of any age or clinical condition receiving healthcare in a hospital, outpatient, or primary care.
I (Intervention):	Open-label placebo (OLP) interventions.
C (Comparison):	Usual care, treatment-as-usual, and standard medical management without a placebo.
O (Outcome):	Subjective outcomes (self-report outcomes, i.e., pain, quality of life, emotional distress, etc.), objective outcomes (physical/physiological measures, i.e., pathological discourse change), safety outcomes (adverse events, discontinuation of treatment).
S (Study):	Clinical trials, or randomized controlled trials (RCTs).
T (Time):	Period from January 2020 to August 2025.

Research Question

In patients receiving healthcare, do OLPs improve outcomes compared with UC or TAU?

Search Strategy and Strings

For all the aforementioned keywords, text words, and MeSH terms, appropriate Boolean operators were used to search literature (Table 2). Only published studies were sought from PubMed, the Cochrane Library, and Scopus. The review restricted the search to only clinical trials or randomized controlled trials (RCTs) written in English and published between the 1st of January 2020, and the 31st of August 2025.

**Table 2:** Search string used on each electronic database ABS: Abstract; KEY: keywords; PUBYEAR: publication year

Electronic Databases	Search Strings
PubMed	Search: (((Clinical Healthcare [All fields] OR Hospital [All fields] OR primary care [All fields]) AND (Open-label placebos [All Fields] OR "placebos" [MeSH Terms] OR placebo, open-label [Text Word])) AND (treatment-as-usual [All Fields] OR standard medical management [All fields] OR usual care [All fields])) AND (subjective outcomes OR pain perception OR objective outcomes) Filters: in the last 5 years, Clinical Trial, Randomized Controlled Trial, English
Scopus	( TITLE-ABS-KEY ( Clinical Healthcare OR Hospital OR primary care ) AND TITLE-ABS-KEY ( Open-label placebos OR "placebos" OR placebo, open-label ) AND TITLE-ABS-KEY ( treatment-as-usual OR standard medical management OR usual care ) AND TITLE-ABS-KEY (Pain OR Symptoms relief OR Outcomes)) AND PUBYEAR > 2020
Cochrane Library	Title abstract keywords (Clinical Healthcare OR Hospital OR primary care AND Open-label placebos OR "placebos" OR placebo, open-label AND treatment-as-usual OR standard medical management OR usual care AND subjective outcomes OR pain perception OR objective outcomes) with Publication Year from 2020 to 2025, in Trials (Word variations have been searched)

Operational Definition of OLPs

OLP is any type of placebo intervention in which patients are clearly told that they are receiving a placebo, and the placebo is given in any form (oral capsule, solution, spray, injection, sham device, or mouthwash). The main objective of OLP is to make use of the placebo effect without deceiving their patient, whereby comparison of the intervention is carried out again. OLP may incorporate C-OLPs, which denote the combination of a placebo with psychological conditioning (e.g., behaviors such as expectation or prior associations to enhance the placebo effect) assigned to different forms of administration (e.g., pills, injections, or other sham devices), as long as the patient knows that he or she is getting a placebo. The most important criterion for inclusion in this definition is that the patient knows that he or she is receiving a placebo, even when psychological conditioning of additional types or different kinds of delivery are involved.

Eligibility Criteria

The eligibility criteria were set relevant to the research objectives, which are presented in Table 3.

**Table 3:** Eligibility criteria TAU: treatment as usual; UC: usual care; OLP: open-label placebo

Inclusion Criteria	Exclusion Criteria
Studies focusing on patients of any age or clinical condition receiving care in healthcare settings (e.g., hospital, outpatient, or primary care).	Studies focusing on healthy individuals or outside clinical or healthcare settings.
Studies focused on open-label placebo (OLP) as primary interventions, where patients are explicitly informed they are receiving a placebo, administered in any form (e.g., oral capsule, solution, injections, sham, mouthwash, spray). The most important criterion for inclusion in this definition is that the patient knows that he or she is receiving a placebo, even when psychological conditioning of additional types or different kinds of delivery are involved. OLP could be done as the only primary intervention.	Studies that focused on only double-blinded or traditional placebos as primary interventions were excluded.
Studies focused on treatment as usual (TAU) and usual care (UC), standard medical management without a placebo, for comparison with an open-label placebo intervention.	Studies focused on interventions other than standard or usual care management were excluded.
Randomized controlled trials and clinical trials were considered exclusively.	Observational studies, cohort study designs, case-control studies, editorials, letters of opinion, and review papers (narrative or literature) were excluded.
Studies that were written in the English language and published in peer-reviewed sources were considered.	Studies published in non-peer-reviewed sources such as preprints, conference abstracts, or articles with insufficient methodological rigor were excluded.
Studies between 1^st^ January 2020 and 31^st^ August 2025 were included.	Studies before 1^st^ January 2020 were excluded.

Selection Process

Studies were screened independently by all authors. Screening conflict was resolved by consensus or by involving a third person who was an independent reviewer. The selection process was carried out using PRISMA 2020 statement guidelines in two phases such as title and abstract screening and full text screening. The Shiny app was used for producing the PRISMA 2020-compliant flow diagram [16].

Data Extraction

Data was extracted independently by all authors with a process to resolve differences. Data was extracted containing information such as author, year, study design, country, type of blinding, sample-related information (mean age, % of females), clinical condition, intervention group, comparator, and findings of each clinical trial using a standardized Excel spreadsheet. The data was extracted by all authors from the included clinical trials. 

Methodological Quality Assessment

Risk of bias (RoB) was assessed using Cochrane RoB 2.0 because selected studies were RCTs. If a study was rated as low RoB in all five domains, it was classified as having overall low RoB. It meant that the evidence is fully consistent and reliable, and it is less likely that future research may alter the evidence. Conversely, if a study demonstrated ambiguity or concerns in one or more domains, it was rated as overall some concerns RoB, meaning that reviewers identified some potential methodological limitations or RoB is not clearly identifiable. A study was rated as high RoB if one or more domains of RoB were identified as significant methodological limitations that may introduce bias and potentially influence the findings of the study [17].

Data Synthesis

Data was combined using a narrative and data-driven synthesis approach. Since the included studies contained statistical data to support the evidence using outcomes related to pain, healthcare burden reduction, reduction in medicine consumption, improved healthcare outcomes, improvement in function, and overall quality of life. The evidence was synthesized while comparing OLP intervention versus UC or TAU to determine the distinguished role of OLP. Moreover, p < 0.05 was taken as significant to determine the statistical significance. The evidence is synthesized using a narrative approach due to the heterogeneity of clinical conditions and outcomes. Owing to the acknowledged heterogeneity, meta-analysis was not performed.

Results

Study Selection Process

**Figure 1:**
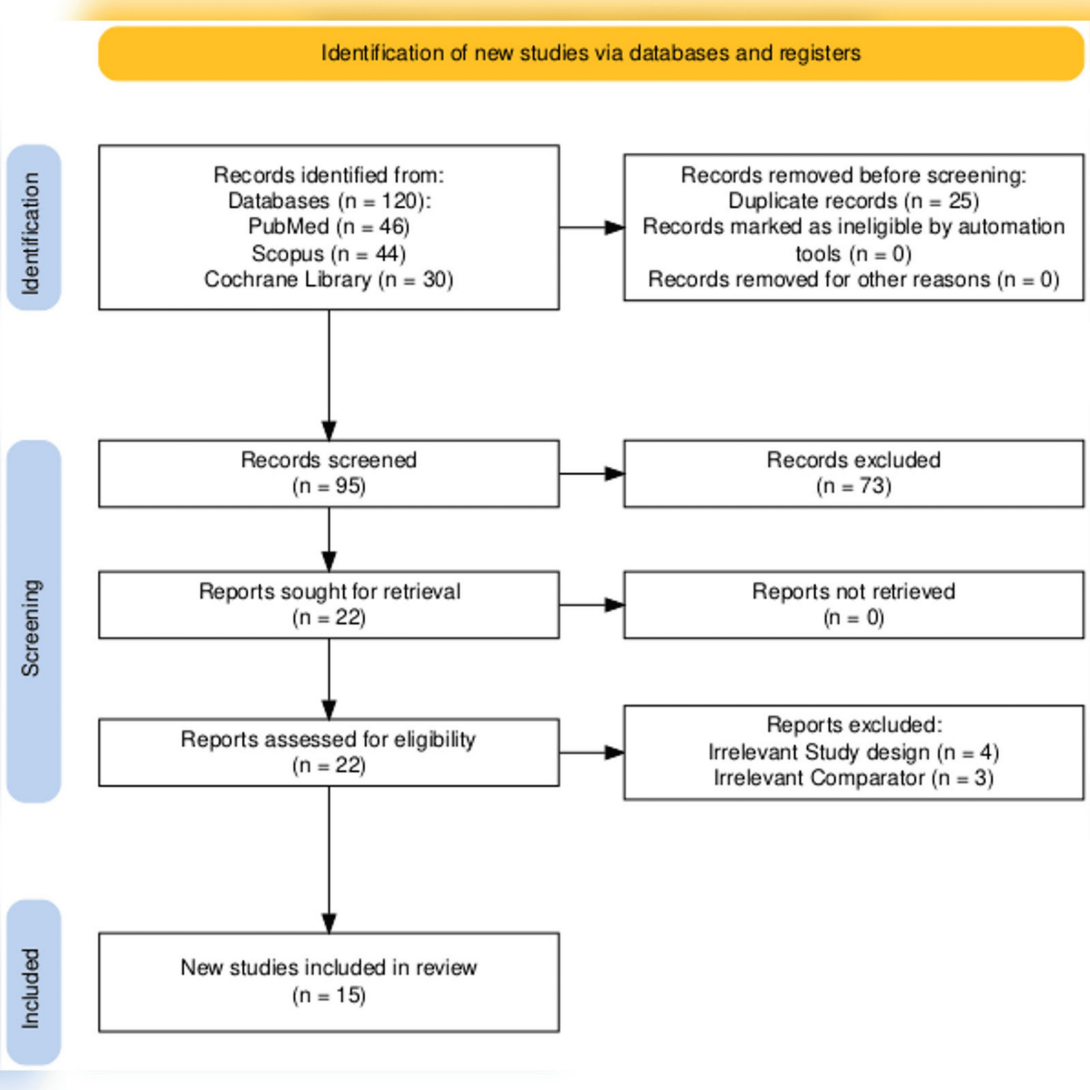
PRISMA flow chart PRISMA: Preferred Reporting Items for Systematic Review and Meta-Analysis

The initial database search yielded 120 studies. After excluding 25 duplicates, the titles and abstracts of 95 studies were screened to determine the relevance. The full text of 22 studies was evaluated, and only 15 met eligibility criteria. Fifteen RCTs were included after an eligibility check following PRISMA guidelines for the study selection process (Figure 1). The majority (eight out of 15 RCTs) were conducted in the USA, two each in Denmark, Germany, and Japan, with only one in the Netherlands.

General Overview

Regarding assessing the sample population, this systematic review synthesized evidence including 1217 adult and adolescent patients with mean ages ranging from 14.1 to 70.7 years. The majority of participants were females, with 55.87% reporting 100% in hot flushes treatment. Additionally, OLPs were also used to treat clinical healthcare conditions such as chronic back or low back pain, opioid use disorder (OUD), knee osteoarthritis (KO), irritable bowel syndrome (IBS), menopausal hot flushes, obesity, and cancer-related fatigue (CRF).

Efficacy of OLPs

To determine the efficacy of OLPs across different clinical healthcare conditions, the 15 RCTs seem divided into two aspects such as eight RCTs showing significant improvement (p < 0.05) and seven RCTs showing insignificant difference as compared to UC or TAU (p > 0.05). Clinical trials on chronic back pain, IBS, menopausal hot flushes, CRF, and seasonal allergic rhinitis showed significant improvements using OLPs [11,12,14,18-21]. However, clinical trials on OUD, KO, chronic low back pain, and COVID-19 symptoms exhibited no significant difference between OLP and UC or TAU [13,22-27]. Hence, there is an inconsistency in clearly determining the efficacy of OLPs across different healthcare conditions.

Effect of OLPs on Patient-Reported and Objective Outcomes

While synthesizing evidence, a consistent theme is observed: eight clinical trials demonstrated that all patient-related outcome measures (PROMs) were significantly improved for pain, hot flushes, COVID-19 symptoms, and seasonal allergic rhinitis [11,12,14,18-21,28]. However, it does not impact clinical conditions or the pathological course of the disease (objective outcomes). This emphasizes that the clinical utility of OLP may be more prominent when the goal is to relieve symptoms rather than to reverse the disease progression. OLPs do not alter the underlying pathology. However, OLPs influence the perception and interpretation in the brain. The characteristics of the included studies are presented in Table 4.

**Table 4:** Characteristics of included studies ADL: activities of daily living; CI: confidence interval; CSMS: combined symptoms and medication score; GLAD: Good Life with Osteoarthritis in Denmark; TNSS: Total Nasal Symptoms Score; OLP: open label placebo; TAU: treatment as usual; UC: usual care; SC: subcutaneous; NR: not reported; MH: monohydrate; MD: mean difference; IA: intra-articular; PIL: patient information leaflet; HCP: healthcare professional; QOL: quality of life; RMDQ: Roland-Morris Disability Questionnaire; NRS: Numeric Rating Scale; IBS: irritable bowel syndrome; IPC: illness perception conversation; RPC: Research Participation Conversation; KOOS: Knee Injury and Osteoarthritis Outcome Score; VAS: visual analogue scale; TUG: Timed-Up-and-Go

Authors (Years)	Country	Study Design	Blinding	Sample (n, mean age, % female)	Clinical Condition	Intervention	Comparator	Findings
Nurko et al. 2022 [11]	USA	Multicenter RCT crossover	Single (outcome assessor blinded)	30, 14.1 + 3.4 years, 80%	IBS	OLP	Control (hyoscyamine pills)	OLP significantly lowered pain (MD, 5.2; 95% CI, 0.2-10.1; P = 0.03), and the control group consumed more pills (MD, 1.8 pills; 95% CI, 0.5-3.1 pills) than OLP.
Schaefer et al. 2024 [12]	Germany	RCT	Single-blinded (outcome assessor)	57, 33.04 ± 14.18, 42.11%	Obesity	Two OLP tablets	TAU (no pills)	Weight loss in OLP = 2.22 ± 1.59 kgs, TAU = 1.26 ± 1.43 kgs (p < 0.05).
van der Palen et al. 2022 [13]	Netherlands	RCT	No	114, 55.3 years, 57%	Asthma	OPL ELLIPTA inhaler + PIL	OPL BREEZHALER + PIL	Fewer patients required HCP instruction with ELLIPTA than BREEZHALER (25% vs. 32%), (OR: 0.25 vs. 0.11) at 95% CI.
Pan et al. 2020 [14]	USA	RCT	Single-blinded (outcome assessor)	100, 54.2 (44–76), 100%	Menopausal hot flushes	OLP	UC (no pills)	OLP reduced the log‑transformed hot flush composite score. (MD change: − 0.32, 95% CI (− 0.43; − 0.21), p < 0.001), 24% females had more than 50% reduction in hot flush (p = 0.02), improved menopause-QOL (p = 0.02).
Ashar et al. 2022 [18]	USA	RCT	No	151 adults, 41.1 ± 15.6 years, 54%	Chronic back pain	OLP SC saline injection	Usual care	Pain intensity 2.84 (OLP) vs. 3.13 (UC). % pain-free/nearly pain-free: 20% OLP vs. 10% UC at post-treatment (P < .001).
Ashar et al. 2024 [19]	USA	RCT	No	101 adults, 40.4 ± 15.4 years, 51.4%	Chronic back pain	OLP nondeceptive injection	Usual care	OLP means pain reduction = 0.61; Hedges' g = 0.45; p = 0.02, at 1 month.
Lembo et al. 2021 [20]	USA	RCT	Single-blinded (outcome assessor)	262, 42.0 ± 18.1, 72.9%	IBS	OLP	DBP, UC	The OLP group showed significantly greater improvement in pain compared to UC (90.6 vs. 52.3, P = 0.038).
Yennurajalingam et al. 2022 [21]	USA	RCT	No	84, 56 + 13, 62%	Cancer-related fatigue (CRF)	OLP—one tablet twice a day	Waitlist (WL) control (no treatment)	The OLP group showed significant improvement in CRF at day 8 (mean change: 6.6 vs. 2.1 in WL, p = 0.016). There was no difference between groups at days 15 and 29 after all patients received OLP.
Belcher et al. 2023 [22]	USA	RCT	Single blinding (outcome assessor)	131, 45.9 + 11.2 years, 63.4%	Opioid use disorder (OUD)	C-OLP (pill + conditioning)	TAU (83.1 mg of Methadone only)	No significant difference between C-OLP & TAU (p = 0.432).
Flowers et al. 2021 [23]	USA	RCT	Single (outcome assessor)	41, 59.1 ± 13.1, 49.5%	Post-spine surgery pain	C-OLP (pill containing microcrystalline cellulose)	TAU of unrestricted opioid-based post-op analgesic regimen: 5 mg oxycodone	Opioid consumption: The COLP group consumed 30% fewer daily morphine milligram equivalents compared to TAU (-14.5 mg, 95% CI (-26.8, -2.2)).
Ginnerup-Nielsen et al. 2024 [24]	Denmark	RCT	Single-blinded (outcome assessor)	103, 70.7 + 7.3 years; 45.6%	Knee osteoarthritis (KO)	OLP (IA-saline injection) + IPC	RPC	(VAS): Both groups showed similar reduction in knee pain (-13.7 for IPC vs. -13.0 for RPC, p = 0.85). KOOS ADL, QOL (p > 0.05).
Henriksen et al. 2023 [25]	Denmark	RCT	Single-blinded (outcome assessor)	206, 68.4 + 8.3 years, 45.6%	Knee osteoarthritis (KO)	OLP-4 IA saline injections	Exercise & education program (GLAD)	KOOS pain: The OLP group showed a mean change of 7.0, while GLAD showed 8.4 (difference = 1.5 points, 95% CI: -2.6 to 5.5, p > 0.05, not significant).
Ikemoto et al. 2020 [26]	Japan	RCT	No	52, 65.3 ± 13.8, 61.5%	Chronic low back pain (CLBP)	OLP + TAU	TAU	No significant intergroup differences in changes in the RMDQ score (p = 0.40), pain-NRS score (p = 0.19), and TUG time (p = 0.98) at week 3.
Onozuka et al. 2024 [27]	Japan	RCT	No	No demographic reported	Mild COVID-19 symptoms	OLP CPC mouthwash	OLP purified water mouthwash	0.05% CPC mouthwash reduced viral titer at 10 minutes post-use (-0.97 log 10 PFU/mL; P = .004) and similarly at 30 and 60 minutes. OLP CPC mouthwash showed better results than OLP alone.
Schaefer et al. 2023 [28]	Germany	RCT	Single-blinded (outcome assessor)	68, 26.71 ± 8.7, 53%	Seasonal allergic rhinitis	OLP (PRoBiotic lactose-MH) Double-blinded OLP (PRoBiotic glucose-MH)	TAU	TNSS, CSMS change scores for the last 12 h; OLP: 1.20 ± 2.41, DBP: 0.25 ± 2.65, TAU: 0.42 ± 2.39; p = 0.014, OLP showed improvements.

Narrative Classification

The eight RCTs of OLP's effectiveness showed among IBS, chronic back pain, CRF, menopausal hot flushes, and seasonal allergic rhinitis patients (p < 0.05). However, Ikemoto et al.'s 2020 [26] study showed non-significant efficacy of OLP to manage chronic low back pain. The study's sample size is underpowered, which may lead to non-significant findings. Moreover, the majority of musculoskeletal structural and acute infectious conditions mostly showed non-significant efficacy of OLP (Table 5). 

**Table 5:** Narrative classification to demonstrate which conditions respond to OLP significantly * indicates that this study sample size is underpowered, which may lead to non-significant results. OLP: open-label placebo

Clinical Conditions With Significant OLP Efficacy	Clinical Conditions With Non-significant Efficacy
IBS [11,20]	Knee osteoarthritis (KO) [24,25]
Chronic back pain [18,19]	Chronic low back pain (CLBP) [26]*
Cancer-related fatigue (CRF) [21]	Opioid use disorder (OUD) [22]
Obesity [12]	Post-spine surgery pain [23]
Menopausal hot flushes [14]	Asthma [13]
Seasonal allergic rhinitis [28]	Mild COVID-19 symptoms [27]

Critical Appraisal of Evidence

**Figure 2:**
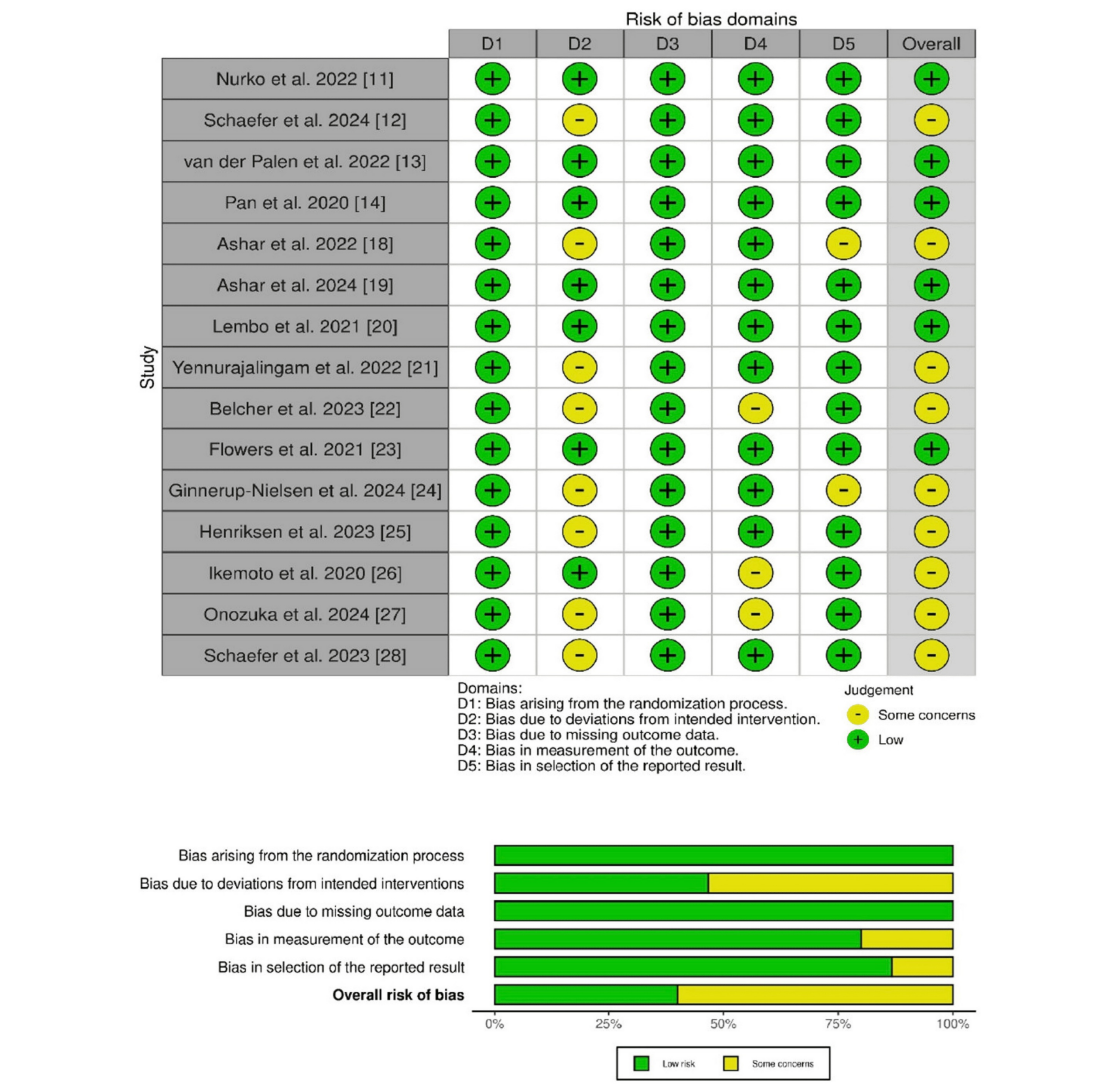
Traffic light and summary plot of Cochrane risk of bias 2.0 among included studies

The critical evaluation of evidence quality using the standard of Cochrane RoB 2.0 reported that six out of 15 studies were reported with overall low RoB, and the majority of nine studies demonstrated some concerns about RoB. The reasoning is explained via domain-wise evaluation. The D2 (bias due to deviation from intended intervention) illustrated some concerns in nine of the studies, explaining that the proper administration of blinding is not followed, which may introduce bias. Although the study design and OLP administration require participant non-blinding, there is a need to apply blinding for the outcome assessor and researcher to prevent bias. It may reduce the ROB in this particular domain. However, the two domains, D3 and D4, also illustrated some concerns for bias due to missing outcome data and bias in the selection of reported results in three and two RCTs, respectively, as illustrated in Figure 2. Therefore, there is a need for further research to make more robust trials, as the traditional double-blind trials will not work, as the mechanism of it is the subject being investigated. Researchers should focus on these domains, particularly D2, to reduce the bias in the synthesis of evidence.

Sensitivity Analysis

**Figure 3:**
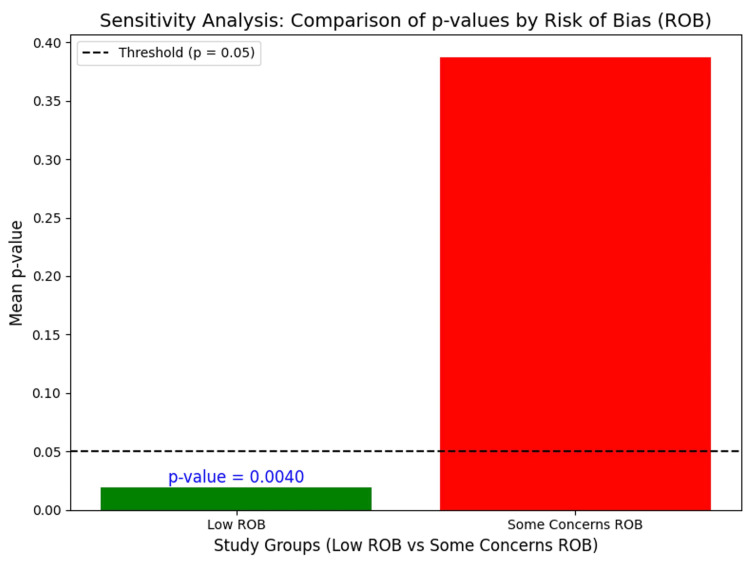
Sensitivity analysis of low versus some concerns ROB studies

In the studies that showed some concern, these studies did not demonstrate significant efficacy of OLP (p > 0.05, red bar), especially in conditions that have greater mechanical or structural components (e.g., knee OA, chronic pain, OUD). However, the authors found the study on seasonal allergic rhinitis to be significant despite some concerns about the ROB, which implies that OLPs may retain efficacy in certain conditions even with some concerns about the ROB. Thus, although there are misgivings of bias that don't support efficacy in OLP in the majority of cases, there might be exceptions in which OLPs might seem to work differently in conditions such as seasonal allergic rhinitis. Overall, the majority of significant studies showed low ROB (Figure 3).

Discussions

This systematic review included 15 RCTs (nine with some concerns of RoB, six with low concerns of RoB) to synthesize evidence. The evidence included 1217 adult and adolescent patients with mean ages ranging from 14.1 to 70.7 years. The majority of participants were females, with 55.87%. The evidence synthesized shows that OLP may have a positive effect, but that varies neither uniformly nor under all conditions. A major finding is the difference between PROMs and objective outcomes. Subjective, self-report outcomes (i.e., pain, quality of life, emotional distress, etc.) rather than objective (physical/physiological) measures have stronger and more consistent positive responses to OLP, indicating an unequal effectiveness of the intervention relating to the subjective and objective impact of the disease. This does not mean that OLPs reverse the process of a latent disease but rather have an impact on the subjective outcomes, such as perception and interpretation of the symptoms. This implies that the main clinical relevance of OLPs can be their use in chronic conditions having a significant subjective element and a main objective of symptom control, as in the cases of pain and fatigue. These findings are all aligned with the published literature [29-31]. 

The clinical trials employed in this review and OLP research as a whole are highly prone to inadequate methodological problems, limiting the confidence of the evidence [29]. The first is that because it is impossible to blind subjects and staff to the intervention, there is a great risk of performance and detection bias. Thus, lack of blinding leads to interrelated biases that are unique to OLP trials. This awareness of obtaining a placebo can cause differentiating missing data, such that patients in the OLP arm are shown to drop out more frequently when they do not sense quick advantages in favor of alternative treatment options. This bias may, in its turn, result in the overstatement of the treatment effect when these dropouts are omitted during the analysis, as the surviving population will now be a biased or unrepresentative subgroup of responders. In addition, even the problem of randomization by itself should be treated with caution. This could be done by concealing the allocation in such a way that the subject who knows their assignment does not affect their conduct or the investigator’s conduct. The guidelines adopted by the Food and Drug Administration (FDA) in this matter are clear and give insights that the subjects in the arms should undergo the same form of treatment, the stringent data collection procedure, and the use of intention-to-treat (ITT) analysis to address the challenges as solutions [32].

OLP has been widely contemplated to be used as an alternative to deceptive placebo use, as it does not infringe on patient autonomy, and it provides transparency [33]. This review is not a discussion of whether deception is acceptable in healthcare but a more refined thinking on a new method of clinical practice [34]. OLP is safe to propose as an effective and practical ethics theory in clinical practice, with moderate consideration of evidence due to some concerns about the ROB. Although the findings present OLP as a low-risk and low-cost treatment that could have of positive impact, especially when contrasted with the higher risks and ethical issues of off-label medication prescription, it still needs to be carefully implemented due to some concerns of RoB. It needs further validation through multicenter RCTs, including long-term follow-up, to determine the certainty of evidence. 

Implications of Findings

Although OLP provides a solution to address an ethical issue of infringing patient autonomy, it gives rise to other, less obvious ethical issues. As an example, unintended harms (external and internal stigma, that some patients might feel like their illness is not taken seriously, or, most importantly, untimely appropriate diagnosis and treatment) are a risk [29]. Effective communication with the patient and the construction of an open and supportive atmosphere are of utmost importance to the success of OLP. Clinicians should educate the patient and establish a strong and transparent connection with the patient [35]. The patient will likely not be a passive recipient but an active participant, which means that the rational decision is made according to the risk-benefit profile following informed consent guidelines (when the patient is assessed to be competent). This also mandates the clinicians to exercise a keen consideration of the possible advantages of OLP in clinical settings, considering patients’ healthcare conditions.

Strengths

The review is conducted following PRISMA guidelines. The methodological quality assessment was performed using the standardized Cochrane RoB 2.0. All evidence was synthesized from recent RCTs, which are considered high-level findings for evidence synthesis. Most of the evidence is based on low and some concerns about RoB; not a single clinical trial of high RoB was reported/used in the review. The evidence is synthesized using the PICOST framework to construct the specific research question and answer it through a narrative approach due to the heterogeneity of clinical conditions and outcomes. Owing to the acknowledged heterogeneity, meta-analysis was not performed. The review emphasized that informed placebos are effective for certain clinical conditions (chronic back pain, IBS, menopausal hot flushes, and CRF). However, further validation is needed.

Limitations and Future Recommendations

Some of the limitations of the review are that the evidence is based on the majority of concerns about RoB clinical trials. The evidence indicated that the methodological limitations of the included clinical trials should minimize the chances of performance and selection bias. The evidence presents methodologically inconsistent efficacy of OLPs in the clinical context. The included trials did not follow up with patients. Only one clinical trial is multicenter, and the remainder were single-center. Hence, given the variability in instruction of administering OLPs, there is a need to devise standardized guidelines or a framework. These frameworks and standardized guidelines for administering OLPs would be recommended by the researchers who will aim to conduct RCTs. Therefore, future researchers are recommended to conduct multicenter clinical trials with more robust methodological rigor, such as the introduction of double-blinding to reduce selection bias and appropriate selection of outcome reporting to minimize bias. Future researchers are encouraged to follow up with patients to observe long-term outcomes.

## Conclusions

The systematic review of recent clinical trial syntheses suggests that OLPs are a potentially ethically viable intervention. It works the most effectively, especially when it comes to symptom management of chronic conditions with a dominating subjective factor (e.g., patient- and psychosomatic-related symptoms or complications). OLPs do not appear to change the pathological course of the disease. Their observed benefits likely stem from a complex interplay between transparent patient-clinician communication and the patient’s capacity to modulate symptom perception. Although it is a benign, low-cost substitute for pharmacotherapy for clinical conditions, the existing evidence is limited by critical methodological biases, such as the majority of included RCTs reporting some concerns about RoB of selection, performance, and attrition bias. Therefore, OLPs should not be used as standalone therapies to replace established, evidence-based treatments for chronic clinical conditions. However, they may be reasonably considered as adjunctive options when conventional treatments have failed, shown limited efficacy, or caused intolerable side effects that lead patients to discontinue standard care. Sensitivity analysis also demonstrated that the majority of studies with non-significant OLP efficacy showed some concerns about ROB, but all low ROB studies showed significant effectiveness. This emphasized that there may be a chance that some concerns of ROB studies may influence the interpretation of the findings. Hence, future researchers should focus on overcoming the aforementioned methodological challenges to present a more resolute clinical value and PROMs for the introduction of OLPs.
